# Psychological and psychosocial determinants of COVID related face covering behaviours: A systematic review

**DOI:** 10.1002/cl2.1422

**Published:** 2024-07-20

**Authors:** Rachel Leonard, Sean R. O'Connor, Jennifer Hanratty, Ciara Keenan, Yuan Chi, Jenny Ferguson, Ariana Axiaq, Anna Volz, Ceri Welsh, Kerry Campbell, Victoria Hawkins, Sarah Miller, Declan Bradley, Martin Dempster

**Affiliations:** ^1^ School of Psychology Queen's University Belfast Belfast UK; ^2^ Centre for Evidence and Social Innovation Queen's University Belfast Belfast UK; ^3^ National Children's Bureau Belfast UK; ^4^ Yealth Network Beijing Yealth Technology Co., Ltd. Shanghai China; ^5^ Centre for Effective Education Queen's University Belfast Belfast UK; ^6^ Centre for Public Health Queens University Belfast Ireland

**Keywords:** COVID‐19, face coverings, knowledge, masks

## Abstract

**Background:**

The COVID‐19 pandemic, caused by the SARS‐CoV‐2 virus, has resulted in illness, deaths and societal disruption on a global scale. Societies have implemented various control measures to reduce transmission of the virus and mitigate its impact. Individual behavioural changes are crucial to the successful implementation of these measures. One commonly recommended measure to limit risk of infection is face covering. It is important to identify those factors that can predict the uptake and maintenance of face covering.

**Objectives:**

We aimed to identify and synthesise the evidence on malleable psychological and psychosocial factors that determine uptake and adherence to face covering aimed at reducing the risk of infection or transmission of COVID‐19.

**Search Methods:**

We searched various literature sources including electronic databases (Medline ALL, Child Development & Adolescent Studies, ERIC, PsycInfo, CINAHL & Web of Science), web searches, conference proceedings, government reports, other repositories of literature and grey literature. The search strategy was built around three concepts of interest including (1) context (terms relating to COVID19), (2) behaviour of interest and (3) terms related to psychological and psychosocial determinants of COVID Health‐Related Behaviours and adherence or compliance with face covering, to capture malleable determines. Searches capture studies up until October 2021.

**Selection Criteria:**

Eligibility criteria included observational studies (both retrospective and prospective) and experimental studies that measure and report malleable psychological and psychosocial determinants and handwashing at an individual level, amongst the general public. Screening was supported by the Cochrane Crowd. Studies titles and abstracts were screened against the eligibility criteria by three independent screeners. Following this, all potentially relevant studies were screened at full‐text level by the research team. All conflicts between screeners were resolved by discussion between the core research team.

**Data Collection and Analysis:**

All data extraction was managed in EPPI‐Reviewer software. All eligible studies, identified through full‐text screening were extracted by one author. We extracted data on study information, population, determinant, behaviour and effects. A second author checked data extraction on 20% of all included papers. All conflicts were discussed by the two authors until consensus was reached. We assessed methodological quality of all included studies using an adapted version of the Joanna Briggs Institute Quality appraisal tool for cross‐sectional studies.

**Main Results:**

Our initial searches yielded 23,587 results, of which 23 were included in this review. The included studies were cross‐sectional in design, came from nine countries and had a combined sample of 54,401 participants. The vast majority of studies had samples from the general public, with five of the studies focusing on specific samples. All included studies considered people over the age of 18. The quality of 10 of the studies was rated as unclear, 10 were rated as low, and 3 rated high risk of bias, predominately due to lack of reporting of recruitment, sample characteristics and methodology. Ten studies were included in the meta‐analysis and 16 in the narrative synthesis. Findings from the meta‐analysis indicated that knowledge of COVID‐19 (0.341, 95% confidence interval [CI] = 0.06, 0.530, *I* ^2^ = 100%) was the malleable determinant most associated with face covering behaviour. Perceived susceptibility of COVID‐19 (*r* = 0.088, 95% CI = −0.004, 0.180, *I* ^2^ = 80%) and COVID‐related worry and anxiety (*r* = 0.064, 95% CI = −0.066, 0.191, *I* ^2^ = 93% had little to no effect on face covering behaviour. In the narrative synthesis, the strongest association was found between perceived benefits and effectiveness of behaviours and mask wearing behaviour.

**Authors' Conclusions:**

Understanding the effects of various malleable determinants on COVID‐related face covering can aid in the development and implementation of interventions and public health campaigns to promote face covering behaviour in potential new waves of COVID‐19 or other respiratory infections. Knowledge of COVID and perceived benefits of face coverings warrant further consideration in future research and policy.

## 1 PLAIN LANGUAGE SUMMARY

### 1.1 Knowledge of COVID and perceived benefits are determinants most associated with face covering behaviour

#### 1.1.1 What is this review about?

Wearing masks is important in preventing the spread of COVID in new waves. Face covering cannot be effective if the majority of society does not adopt this behaviour. So, it's crucial to understand the changeable factors that affect this behaviour.

This review was interested in psychological or psychosocial determinants of face covering behaviour. To be included, determinants were malleable factors (factors that could be changed).

#### 1.1.2 What is the aim of this review?

This Campbell systematic review examines the factors that influence wearing face masks. The review summarises evidence from 23 studies.

#### 1.1.3 What are the main findings of this review?

##### What studies are included?

This review studied various factors that influence wearing face masks during COVID‐19. We reviewed 23 studies. We analyzed the data within 10 studies and summarized the data in 16 studies narratively. The research was done in 9 countries during the COVID‐19 pandemic. Many studies had unclear quality. Three had methodological weaknesses, like lack of detail, such as who their sample was and how they measured face covering.

##### What determinants were associated with face covering?

Determinants that were associated with face covering included, knowledge of COVID, and perceived benefits. COVID concerns and worries did not affect face covering behaviour.

#### 1.1.4 What do the findings of this review mean?

We need more studies on how knowledge of COVID and perceived benefit of face covering affects face covering behaviour. Future policies that try to prevent the spread of COVID or other respiratory diseases might also consider these factors.

#### 1.1.5 How up‐to‐date is this review?

The review authors used search methods to find studies until October 2021.

## 2 SUMMARY OF FINDINGS

Summary of findings 1

Summary of findings

**Table:** 

**Determinant**	**Effect size**	**95% CI**	***Q***	***I* ^2^**	***τ* ^2^**	***k***
Knowledge of COVID	*r* = 0.341*	0.06, 0.530	940.834	100%	0.091	5
Worry and anxiety about COVID	*r* = 0.064	−0.066, 0.191	28.028	93%	0.012	3
Perceived susceptibility	*r* = 0.088	−0.004, 0.180	10.237	80%	0.005	3

Abbreviations: CI, confidence interval; *I* ^2^, percentage of variability due to between‐study heterogeneity; *k*, number of effect sizes; *Q*, test for heterogeneity; *r*, correlation; *τ* ^2^, random effects variance component.

*p < 0.05.

## 3 BACKGROUND

### 3.1 The problem, condition or issue

Severe acute respiratory coronavirus 2 (SARS‐CoV‐2) emerged in late 2019 and spread rapidly around the globe (Cucinotta & Vanelli, 2020; Wu et al., 2020). The pandemic of COVID‐19 disease, caused by SARS‐CoV‐2, has resulted in short and long‐term illness, deaths and societal disruption. Societies implemented control measures to reduce the transmission of the virus. Individual behaviour change is crucial to the success of these measures through reducing the frequency of social contacts, mitigating the risk of those social contacts and reducing the amount of time that infectious people are in contact with others whom they may infect. Vaccine programmes were introduced in December 2020 but even in this context, with waning immunity and the evolution of new variants, behavioural measures to reduce the spread remain vital (Girum et al., 2021; Michie & West, 2020).

The behaviours to reduce the risk of catching or spreading SARS‐CoV‐2 including: handwashing or use of hand sanitiser, wearing masks or face coverings, physical distancing, social distancing, isolation or quarantine, respiratory hygiene, cleaning surfaces, avoiding touching the ‘T‐zone’ (mouth, nose and eyes) (Elder et al., 2014) as well as other composite measures that include these behaviours.

The evidence for the effectiveness of these measures has been established during previous pandemics of similar serious viral respiratory infections such as pandemic Influenza A (H1N1), SARS and MERS (Flumignan et al., 2020; Jefferson et al., 2023; Seto 2003; Warren‐Gash et al., 2013; Webster et al., 2020; West et al., 2020). It is important to synthesise evidence from the COVID‐19 pandemic that may be applied to future pandemics of influenza and other serious respiratory infectious diseases.

### 3.2 Exposure/determinants

The exposure in this review was psychological or psychosocial determinants of face covering. To be included determinants were malleable factors that could, theoretically, be changed by a public health intervention.

### 3.3 Why it is important to do this review

Face covering cannot be effective on a societal level if it is not adopted widely and consistently. Variables such as individual health beliefs, social support, culture, and social norms can all influence the likelihood of someone undertaking and maintaining health behaviours such as face covering. To develop appropriate public health interventions to improve uptake and adherence to face covering, including effective messaging, it is important to understand the malleable factors that influence this behaviour. We identified and examined all existing research evidence that described a relationship between any malleable factor or determinant (or those that can be most effectively targeted as part of public health interventions) and face covering in the context of SARS‐CoV‐2.

In any future severe viral outbreaks, health‐protective behaviours, such as face covering, will be vital to reducing risk of infection and transmission. Non‐pharmaceutical interventions that are designed to improve the uptake and adherence to protective behaviours are essential in an outbreak, and in particular when vaccines and treatments are not yet established. The effectiveness of these behaviour change interventions will be determined, to some extent, by how they address the psychological and psychosocial variables that influence behaviour. To optimise public health intervention, we need to know which specific variables are most likely to influence the target behaviours, such as face covering, in this context. Evidence gathered in the context of COVID‐19 can inform who, when and under what circumstances people do or do not adopt recommended preventive behaviours.

There are a number of related published and ongoing reviews on individual determinants of COVID‐19 health‐related behaviours but none with the broad scope of this review. Using robust search, retrieval, and methodological approaches to minimise potential sources of bias, this review examines the existing and emerging evidence on determinants of face covering in the context of the COVID‐19 pandemic.

### 3.4 Overview of the COHeRe project

COHeRe is a UKRI funded project https://www.qub.ac.uk/schools/psy/Research/OurResearchThemes/HealthWelfareClinicalPsychology/COHeRe made up of a team with substantial expertise in systematic reviews, health behaviour and infectious diseases. The overall aim of the project was to identify, synthesis, and examine evidence on determinates of COVID‐19 health‐related behaviours. The specific behaviours of interest were as follows:

Handwashing
Wearing masks/face coverings
Physical Distancing (maintaining the recommended distance from others when physically present (West et al., 2020))
Social Distancing (minimising social contact with those outside of your own household [West et al., 2020])
Isolation/quarantine
Respiratory hygiene
Cleaning surfaces
Avoiding t‐zone

Other composite measures that include the above.

During Phase 1 of the project a rapid review was conducted, which examined determinants of protective behaviours during COVID‐19 and during previous outbreaks of similar serious respiratory infections, for example, SARS, MERS and H1N1 (swine flu) (Hanratty 2021). Of the 233 studies included in the rapid review, 54 were conducted in the context of COVID‐19, while the remainder were conducted in the context of other respiratory infections. Over the course of conducting the rapid review, it became apparent that the evidence base examining determinants in the context of COVID‐19 was rapidly expanding and further identification and examination was needed of this new evidence.

On this basis, further funding was secured to conduct Phase 2 of the project, which identified and mapped the existing evidence (published and unpublished between January 2020 and October 2021) on malleable and non‐malleable psychological and psychosocial factors that determine uptake and adherence to behaviours aimed at reducing the risk of infection or transmission of COVID‐19 (Hanratty 2022; Hanratty 2023). As of 1st June 2022 the Evidence and Gap Map (EGM) includes 1034 records https://eppi.ioe.ac.uk/eppi-vis/login/open?webdbid=188.

This current review is the final phase of the wider project. Based on those studies included in the EGM we further examined these, through a series of systematic reviews examining which malleable determinants (or those that can be most effectively targeted as part of public health interventions) are more closely associated with uptake and maintenance of individual protective behaviours. This current review examines the protective behaviour of handwashing, however is part of a series of reviews considering the eight other behaviours of interest.

## 4 OBJECTIVES

We intended to identify and synthesise the existing evidence on malleable psychological and psychosocial factors that determine uptake and adherence to face covering that can reduce the risk of infection or transmission of COVID‐19.

## 5 METHODS

### 5.1 Criteria for considering studies for this review

#### 5.1.1 Types of studies

This systematic review contains studies that quantify the relationship between a malleable determinant and face covering. Included study designs consisted of observational studies (both retrospective and prospective) and experimental studies that measure and report malleable psychological and psychosocial determinants and face covering at an individual level. We did not include narrative reviews, modelling studies, letters, editorials, opinion pieces, news, commentaries, or any other publications that did not report primary data.

#### 5.1.2 Types of participants

The population of interest is members of the general public, of any age. Within the group of studies of the general public, we included studies on specific groups of people that may be at increased risk of catching the virus for example, people who work in essential retail services. Similarly, we included studies of specific patient groups at increased risk of becoming seriously ill if infected, for example, those with existing chronic respiratory disorders. However, we did not include studies on health care workers (HCWs), defined as someone who works in a hospital or health care setting or delivers health care in the community. This population typically have, or should have additional knowledge, training and resources to support the adoption of behaviours to mitigate against the increased risk of exposure to infectious diseases. A rapid review on barriers and facilitators to HCWs adherence to infection prevention and control guidelines has been published (Houghton 2020). For those studies that included both HCWs and the public, were only included if data on the public is presented separately from data on healthcare workers.

#### 5.1.3 Exposure/determinants

The exposure in this review was psychological or psychosocial determinants of face covering. To be included determinants were malleable factors that could, theoretically, be changed by a public health intervention.

We developed 10 categories of determinants for phase 2 of this project. These included, behaviour, cognition, demographics, disease, emotions, health status, information, intervention, knowledge and other. Each category was divided into subcategories of various determinants. As above, only malleable determinants were included in this review. Therefore, the following determinants were included:

Cognition was broken down into six subcategories: thoughts or perceptions about the protective behaviours; about COVID‐19; motivations; social cognition (e.g., perceived social norms); cognitive capacity indicating a person's ability to understand or retain information; ‘other’ to capture any other cognitive determinant that did not fit into the previous five subcategories.

Emotions captured determinants related to feelings about the disease (such as worry and anxiety about COVID) and ‘other’ emotion‐related determinants for example general emotional state or mood.

Information included seeking and consuming information, the quality or source of information, and determinants related to public health messaging, for example, message content or framing.

Knowledge included determinants relating to knowledge about protective behaviours, knowledge about the disease and any other types of assessed knowledge, such as knowledge of regulations or knowledge of vaccines.

Other was the final category of determinants and includes any determinants that did not fit within the previous broad categories. This was divided into subcategories of beliefs for example political beliefs, social (e.g., social capital, social networks), practical resources such as access to masks, paid sick leave, time included time since the outbreak began, cultural determinants such as collectivist versus individualist cultures, and a final ‘other’ subcategory for any remaining determinant that did not fit into the previous subcategories.

The determinants of behaviour, demographics, disease, and health status were not included as these were categorised as non‐malleable. We also did not include studies that examined interventions as a determinant of face covering as this will be analysed in a separate review.

Comparators were the absence of the determinant (compared to its presence) or, where a determinant is presented as a continuous measure, then analysis will be based on correlation between face covering and determinants.

We included studies that measured determinants at an individual level and group level, for example, country‐level data on the number of cases.

We included studies on self‐reported or observed determinants. Self‐reports included actual or perceived determinants, for example ‘risk of contracting the virus’ could be measured by quantifying actual risk based on individual circumstances and behaviour or through self‐reported perceived risk.

#### 5.1.4 Types of outcome measures

While our searches sought to identify evidence on commonly recommended behaviours to mitigate human‐to‐human spread of COVID‐19 as described by West et al. 2020, this current review focuses on face covering only. We define face covering as, wearing any type of mask or face covering. This can include medical grade masks, face shields, homemade masks or covering face with a scarf (West et al., 2020).

We included studies on actual face covering behaviour, through self/other report and/or observation, measured at the individual level. We excluded studies that measured intended behaviour or hypothetical behaviour.

##### 5.1.4.1 Primary outcomes

The primary outcome of this review was face covering. No secondary outcome was considered.

### 5.2 Search methods for identification of studies

To ensure that the literature contained in the review was relevant and useful to key stakeholders, it was important that the literature retrieval methods followed high‐quality standards and all searches were conducted and reported following Campbell Collaboration guidelines (White et al., 2020).

Information retrieval specialist author (CK) developed and piloted a search strategy with input from clinical and behaviour change expert authors (DB and MD). This strategy was further refined by CK following expert advice from a Campbell information retrieval specialist during the editorial/peer review of the protocol. Searches capture studies up until October 2021.

The search strategy was built around three concepts of interest;

(1) Context (terms relating to COVID‐19). For concept one, we used an innovative and tested COVID‐19 search strategy was developed for use by NICE information specialists and was updated as recently as 21 June 2021 (Levay & Finnegan, 2021). An example of the search string was piloted in Medline (Ovid) and is presented in Table 3.

(2) Behaviours of interest.

(3) Terms related to psychological and psychosocial determinants of COVID Health‐Related Behaviours and adherence or compliance with recommended behaviours, to capture both malleable and non‐malleable determinants.

For concept 2 and 3 the terms used were based on those used in the rapid review (Hanratty 2021) which itself was informed through consultation with the Behaviour Change Group formed in response to COVID‐19 by the Public Health Agency, Northern Ireland. The terms were then piloted and refined in two databases, with unique terms added and redundant or duplicate terms removed (Table 1).

**Table 1:** Medline (Ovid) search strategy.

Ovid MEDLINE(R) ALL <1946 to 3 September 2021>		
1	SARS‐CoV‐2/or COVID‐19/	103,591
2	(corona* adj1 (virus* or viral*)).ti,ab.	2364
3	(CoV not (Coefficien* or ‘co‐efficien*’ or covalent* or Covington* or covariant* or covarianc* or ‘cut‐off value*’ or ‘cutoff value*’ or ‘cut‐off volume*’ or ‘cutoff volume*’ or ‘combined optimi?ation value*’ or ‘central vessel trunk*’ or CoVR or CoVS)).ti,ab.	51,911
4	(coronavirus* or 2019nCoV* or 19nCoV* or ‘2019 novel*’ or Ncov* or ‘n‐cov’ or ‘SARS‐CoV−2*’ or ‘SARSCoV‐2*’ or SARSCoV2* or ‘SARS‐CoV2*’ or ‘severe acute respiratory syndrome*’ or COVID*2).ti,ab.	181,470
5	or/1‐4	187,096
6	limit 5 to yr = ‘2020‐Current’	173,962
7	(6 and english.lg.) not (letter or historical article or comment or editorial or news).pt. not (Animals/not humans/)	134,173
8	(Mask or masks or face?mask* or Face cover*).ti,ab.	42,975
9	(face adj2 (shield or shields)).ti,ab.	414
10	(((Hand or hands) adj2 hygiene) or Handwash* or (Wash* adj2 hand*)).ti,ab.	11,132
11	(hand adj1 clean*).ti,ab.	256
12	(hand adj2 saniti*).ti,ab.	683
13	(hand adj2 disinfect*).ti,ab.	783
14	Respiratory hygiene.ti,ab.	79
15	Respiratory etiquette.ti,ab.	27
16	((cough* or sneeze*) and (sleeve or arm or elbow or tissue or etiquette)).ti,ab.	2752
17	(tissue and (dispose or disposal or bin or hygiene)).ti,ab.	3414
18	universal hygiene.ti,ab.	10
19	Social Isolation/or Patient Isolation/	19,284
20	(self‐isolate or self‐isolation or self‐isolating).ti,ab.	724
21	(mass adj2 (behav* or gather*)).ti,ab.	1690
22	(social distance or social distancing).ti,ab.	6625
23	stay at home.ti,ab.	1465
24	stay home.ti,ab.	314
25	((work* adj2 home) or telecommute or telework* or (remote* adj2 work*)).ti,ab.	5262
26	(Physical adj2 distanc*).ti,ab.	2595
27	(touch* and (mouth or mouths or face or faces or nose or noses or t‐zone)).ti,ab.	1635
28	disinfect*.ti,ab.	31,760
29	lockdown.ti,ab.	8167
30	quarantine.ti,ab.	7821
31	(nonpharmaceutical or non‐pharmaceutical).ti,ab.	1831
32	(school closure or close school* or school closing).ti,ab.	389
33	or/8‐32	140,404
34	limit 33 to yr = ‘2020‐Current’	34,955
35	(34 and english.lg.) not (letter or historical article or comment or editorial or news).pt. not (Animals/not humans/)	31,455
36	7 and 35	20,298
37	exp Knowledge/	12,323
38	exp Health knowledge, Attitudes, Practice/	119,567
39	(Knowledg* or Personal* or Attitude* or Practice* or Habit* or belie* or Behav* or Need* or prevent* or Compliance or comply* or complied or Perception* or Protect* or Predict* or view* or barrier* or facilitator* or readiness or prepar* or ability* or insight or proficien* or procedur* or adher*).ti,ab.	10,617,318
40	or/37‐39	10,635,825
41	7 and 35 and 40	14,859

#### 5.2.1 Electronic searches

Based on the Queens's University Belfast database subscriptions, we searched the following key information sources to locate relevant primary research:

Medline ALL (Ovid)
Child Development & Adolescent Studies (EBSCOhost)
ERIC (EBSCOhost)
PsycInfo 1806‐present (OVID)
CINAHL Plus (EBSCOhost)
Web of Science Core Collection (the QUB subscription includes SCI‐expanded, SSCI, A&HCI, CPCI‐S, CPCI‐SSH, ESHI)

To locate relevant secondary research for inclusion in the EGM, we searched the following information resources:

The Social Care Institute for Excellence (SCIE)
The Cochrane Library
Epistemonikos Covid‐19 evidence platform
Norwegian Institute of Public Health living maps
EPPI – centre
COVID‐END

#### 5.2.2 Searching other resources

We searched for Grey literature across multiple sources. Grey literature is that which is not published, not peer reviewed, and not easily accessible. Sources of grey literature are varied and include government reports, privately and publicly funded research, conference proceedings, working papers, and posters. Some grey literature sources are captured in the Web of Science search, these include:

Conference Proceedings Citation Index‐ Science (CPCI‐S)—1990‐present
Conference Proceedings Citation Index‐ Social Science & Humanities (CPCI‐SSH)—1990‐present

We attempted to locate additional grey literature by searching sources such as the following:

Google Scholar (We will search https://scholar.google.com/ using an incognito browser and the following strategy: (coronavirus| ‘2019 nCoV’| ‘2019 novel’| ‘2019 nCoV’| ‘2019 nCoV’| CoV |‘COVID 19’ |COVID19| ‘COVID 19’| ncov |‘SARS CoV2’| ‘SARS CoV 2’|‘severe acute respiratory syndrome Coronavirus 2’) (Psychological|Psychosocial)(behavior|behaviour) we will limit returns by ‘Since 2020’ filter and sort remaining records by relevance. We downloaded the first 1000 articles (which is the upper limit set by google) using Harzing's Publish or Perish software.
clinicaltrials.gov
ISRCTN Registry (https://www.isrctn.com/)
WHO International Clinical Trials Registry Platform (ICTRP) (https://www.who.int/clinical-trialsregistry-platform/the-ictrp-search-portal)
And by contacting and reviewing the information of the following key organisations in the UK with proven experience on the topics related to this project:
King's Fund (https://www.kingsfund.org.uk/)
National Institute for Health Research (https://www.nihr.ac.uk/) NHS Evidence (https://www.evidence.nhs.uk/)

We considered searching ProQuest dissertations and theses, however, we assessed that it was unlikely that any relevant doctoral theses would be complete and available in the timeframe of the virus.

We conducted a search of reference lists of previous reviews and eligible articles to identify any additional studies not identified through the electronic search. Finally, when we compiled a list of included studies, we contacted key experts in the field via email (categorised as ‘key’ if they have published five or more included studies) to ask whether they were aware of any unpublished or ongoing research that might not have been easily accessible to the research team.

To locate additional relevant grey literature for inclusion in the EGM, we searched for ongoing or unpublished reviews via:

PROSPERO,
Figshare and the
Open Science Framework (OSF).

Any ongoing reviews were checked again before completion of the project and if still unpublished were excluded from the map.

#### 5.2.3 Search limits

Due to the limited language skills of the review team, we only included studies published in English.

We limited our search to exclude opinion pieces, letters, editorials and unpublished reports in databases where these limits are supported (see Table 3: line 7 and 35). We did not use database limiters for studies on humans only as we found these limiters excluded a substantial number of potentially relevant papers not indexed as ‘human’ studies. Instead, we have opted to use an adaptation of the Cochrane search filter for human studies (line 7 and 35).

We included only those studies which were conducted during the ongoing COVID‐19 pandemic. We included studies from January 2020 until the date of the final search.

### 5.3 Data collection and analysis

#### 5.3.1 Selection of studies

All search results were first screened on titles and abstracts against the eligibility criteria by three independent screeners (each title and abstract was screened three times independently). Screening at this first stage was supported by the Cochrane Crowd. We retrieved a full‐text copy of all potentially relevant studies during the title and abstract screening.

Following this, all potentially relevant studies were screened independently by two reviewers from the research team at full‐text level. All conflicts between screeners were resolved by discussion between the core research team.

#### 5.3.2 Data extraction and management

All data extraction was managed in EPPI‐Reviewer software. All eligible studies, identified through full‐text screening were extracted by one author, who also completed the quality appraisal assessment. Any studies identified as ineligible during data extraction stage were listed as ‘excluded’. A second author checked the data extraction and risk of bias assessments on 20% of all included papers. No systematic data extraction errors were discovered through this quality check, therefore no more than 20% of all extraction was checked. The two people who completed the data extraction for each study discussed any discrepancies until they reached a consensus or, referred to a third author to make a final decision. In addition, the research team met on a weekly basis to discuss extraction and discrepancies, in aid coherence to the extraction protocol. Where data was not available or was missing within an included study, the research team attempted to obtain or clarify data from the relevant authors.

Extracted information included (Supporting Information S1: Appendix 1):

**Study information:** Author, year, country, study design, when the study was conducted, sample size.
**Population:** description of the population, age, sex.
**Exposure:** determinant measured, description of the determinant, who measured the determinant, type of measurement (observation, self‐reported, etc.), direction and quality of the scale.
**Outcome:** behaviour measured, description of the behaviour, who measured the behaviour, type of measurement (observation, self‐reported, etc.), direction and quality of the scale.
**Effects:** Narrative description of the finding, effect size information or sufficient numerical data to allow us to calculate the effect size.

#### 5.3.3 Quality appraisal

The JBI tool for cross‐sectional studies was used to assess the quality of included studies (The Joanna Briggs Institute 2017; The Joanna Briggs Institute 2020). After piloting the JBI tool on some known studies we decided to modify the tool to ensure that they are fit for our purposes (Supporting Information S1: Appendix 2). We changed the wording of the second item ‘were the study subjects and the setting described in detail’ to ‘was the sample included in the study representative of the population of interest?’ to assess whether or not the sample was representative of the population of interest. We also changed the wording slightly, replacing condition and exposure with behaviours of interest and determinants respectively.

The eight questions were answered with either ‘yes’, ‘no’, or ‘unclear’. For the questions on scale validity and reliability, we indicated whether a single item or multiple item scale was used and whether or not this was reliable and valid. Each study was rated either low, high or unclear risk of bias through adding up the total number of items answered ‘yes’. For example, >70% yes = Low Risk of Bias, 50%–70% yes = Unclear Risk of Bias, and <50% ‘Yes’ = High Risk of Bias.

#### 5.3.4 Measures of treatment effect

We extracted data on the relationship between face covering and determinants of that behaviour. Outcomes were reported in both dichotomous and continuous data. The meta‐analysis was performed using Comprehensive Meta‐Analysis Version 4 (Borenstein 2022), and conducted mostly using correlation coefficients (*r*), as that was the effect size statistic most commonly reported in the papers. Therefore, data was extracted that allowed us to convert or calculate *r*. For example, where summary statistics were not presented, we extracted data such as means and standard deviations that allowed us to calculate a standardised mean difference that was then converted to *r*. Effect sizes were interpreted according to thresholds suggested by Cohen 1988: weak (*r* = 0.1), moderate (*r* = 0.3), and strong (*r* = 0.5).

#### 5.3.5 Assessment of heterogeneity

Heterogeneity was assessed first, through visual inspection of the forest plot and checking for overlap of confidence intervals and second through the *Q*, *I* ^2^, and *τ* ^2^ statistic. Investigation of the source of heterogeneity is addressed in data synthesis section.

#### 5.3.6 Data synthesis

Given the diverse range of behaviour and determinant relationships examined across the included studies, we used random effects models, using inverse‐variance estimation, for meta‐analysis of correlation coefficients. We conducted separate meta‐analyses for each determinant of the behaviour of interest, face covering. Data was synthesised based on the follow criteria:

- Determinants were grouped based on previous mapping (Hanratty 2023).
- Determinant groups were included in the meta‐analysis if they included data that was suitable for meta‐analysis (i.e., unadjusted data) and there was a minimum of three data points.
- We excluded adjusted estimates from meta‐analyses as there is considerable variation in the covariates used to adjust these estimates across studies and, therefore, we judged that the adjusted estimates were not suitable for statistical aggregation.
- Data that was not suitable for meta‐analysis was synthesised narratively.

#### 5.3.7 Treatment of qualitative research

The review does not include qualitative research.

## 6 RESULTS

### 6.1 Description of studies

#### 6.1.1 Results of the search

**Figure 1:**
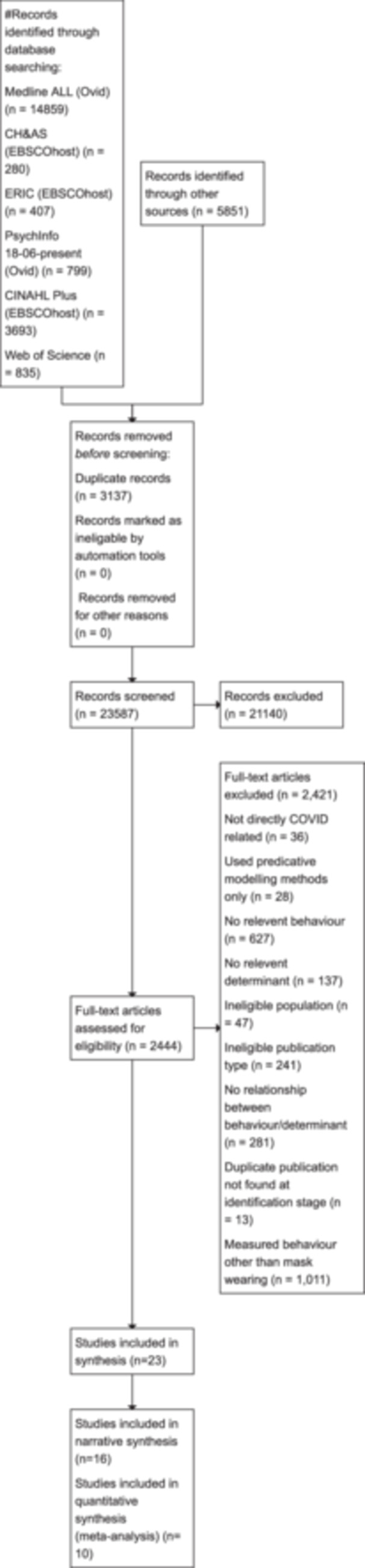
PRISMA flowchart.

As seen in Figure 1, our searches yielded a total of 23,587 results. After screening out titles/abstracts we were left with 2444 results. Of these 2444 studies 2421 were excluded. Reasons included, being directly COVID‐related, using predicative modelling methods, not relevant behaviour (including behaviours like hand washing, distancing included in our other reviews reported elsewhere) or determinant, ineligible population or publication, no relationship measured between behaviour and determinant or a duplicate not found at initial screening stage. Following full‐text screening of these results yielded 23 eligible studies.

#### 6.1.2 Included studies

A total of 23 studies were included in this review. Of these 23 studies, 21 used a cross sectional design, with 2 using a longitudinal design (Goldberg 2020 [Goldberg et al., 2020]; Milad 2021 [Milad & Bogg, 2021]). The 23 studies came from nine different countries, with the majority coming from the USA (*n* = 4) and China (*n* = 3). Other countries included, Poland, Croatia, South Korea, Germany. One study had data from across multiple countries (Varghese 2021 [Varghese Nirosha et al., 2021]). Full details of included studies can be found in Table 2.

**Table 2:** Demographics of included studies.

Study	Country	Study design	Describe population	Age	Sex ‐number of women/girls	Sex ‐number of men/boys	Overall quality rating
Azlan (2020)	Malaysia	Cross sectional	General public (*n* = 4850)	*M* = 34 years (SD = 11.2, range = 18–73)	2808	2042	Low risk of bias
Brankston (2020)	Canada	Cross sectional	General public (*n* = 4981)	18‐29 years (15.7%)	50.6%	49.1%	Low risk of bias
Davis (2021)	USA	Cross sectional	University students (*n* = 595)	*M *= 24.86 (SD = 10.62)	441 (73.4%)	143 (23.8%)	Unclear risk of bias
Goldberg (2020)	USA	Longitudinal	General public (*n* = 3933)	*M* = 47 years (April 3–4) *M* = 46 years (April 5–7)	53% (April 3–4) 56% (April 5–7)	47% (April 3–4) 44% (April 5–7)	Low risk of bias
Gray (2020)	New Zealand	Cross sectional	General public (*n* = 1015)	23.3% of respondents were aged 25–34 years	592 (58.3%)	416 (41%)	Low risk of bias
Hao (2021)	USA	Cross sectional	General public (*n* = 2047)	Not reported	Not reported	Not reported	Low risk of bias
Iqbal (2021)	Pakistan	Cross sectional	General public (*n* = 1789)	*M* = 23.4 years (SD = 8.23)	949 (53.05%)	840 (46.95%)	High risk of bias
Jang (2020)	Korea	Cross sectional	General public (*n* = 2002)	19–29 (18.3%)	505	497	Low risk of bias
Kowalski (2020)	Poland	Cross sectional	General public (*n* = 507)	*M* = 44.07 (*SD* = 14.41)	253	254	Low risk of bias
Krupic (2021)	Croatia	Cross sectional	General public (*n* = 500)	*M* = 27.92 (SD = 9.30).	341 females	159 males	Low risk of bias
Lee (2020)	South Korea	Cross sectional	General public (*n* = 973)	*M* = 46.41 (14.94)	50% 487	50% 486	Low risk of bias
Mahalik (2021)	USA	Cross sectional	Men (*n* = 596)	(*M* = 31.33, SD = 10.59)		100%	Unclear risk of bias
Milad (2021)	USA	Longitudinal	General public (*n* = 451)	*M* = 45.40 (SD = 15.78)	Not stated	Not stated	Unclear risk of bias
Mo (2021)	China	Cross sectional	Pregnant women (*n* = 4087)	Two‐thirds (2743/4087, 67.1%) of the participants were aged 30 years or less.	4087	No men in sample	Unclear risk of bias
Mousavi (2021)	Afghanistan	Cross sectional	General public (*n* = 450)	17–26 years (65.8%)	138 (28.4%)	322 (71.6%)	Unclear risk of bias
Muslih (2021)	Indonesia	Cross sectional	General public (*n* = 1033)	17–29 years: 552 (53.4%) >30 years: 481 (46.6%)	649 (67.2%)	339 (32.8%)	Unclear risk of bias
O'Brien (2021)	USA	Cross sectional	General public (*n* = 450)	*M* = 36.68 (range 18–76)	38%	62%	Unclear risk of bias
Rieger (2020)	Germany	Cross sectional	University students and staff (*n* = 206)	*M* = 26 (range 14–62)	66%	Not reported	Unclear risk of bias
Stosic (2021)	USA	Cross sectional	General public (*n* = 1629)	Not reported	Not reported	Not reported	Low risk of bias
Tran (2020)	France	Cross sectional	Adults with chronic conditions (*n* = 7169)	*M* = 46.1 (14.7) years	5616 (78.3%)	Not reported	Unclear risk of bias
Varghese (2021)	Europe: Germany, UK, Denmark, Netherlands, France, Portugal, Italy	Cross sectional	General public (*n* = 7500)	Not reported	Not reported	Not reported	High risk of bias
Zhong (2020)	China	Cross sectional	General public (*n* = 6910)	*M* = 33.0 years (SD 10.7, range: 16–87)	65.7%	34.3%	High risk of bias
Zhou (2021)	China	Cross sectional	Residents of Wuhan City (*n* = 728)	36–50 years (44.0%)	416 (57.1%)	312 (42.9%)	Unclear risk of bias

There was a total of 54,401 participants across the 23 studies, ranging from 7500 (Varghese 2021) to 206 (Rieger 2020 [Rieger, 2020]). The vast majority of studies had samples from the general public (*n* = 18), with 5 of the studies focusing on specific samples. These included; university students (Davis 2021 [Davis Robert et al., 2021]; Rieger, 2020), men (Mahalik 2021), pregnant women (Mo 2021 [Mo Phoenix Kit et al., 2021]), adults with chronic conditions (Tran 2020 [Tran & Ravaud, 2020]).

All studies included participants over 18 years old. Reporting of age varied between studies, some providing mean age of participants, others providing percentage of age ranges and some not reporting age (Hao 2021 [Hao et al., 2021]; Stosic 2021 [Stosic Morgan et al., 2021]; Varghese 2021). For those studies that did report on age of participants, the average age was 36.58 years.

##### Reported outcome

Studies varied in their approaches to measuring mask wearing. Measures ranged from scales (e.g., Milad 2021; Mo 2021) to single items (e.g., Are you wearing a face mask in response to the coronavirus, Hao 2021). In eight of the studies, mask wearing was measured through single items, with yes or no responses (e.g., I wear a mask when in public, Kowalski 2020 [Joachim et al., 2020]), while others used Likert scales to measure frequency of use (i.e., mask wearing ‐ never, sometimes, often, Lee 2020 [Lee & You, 2020]). Some studies measured adherence to specific mask wearing guidance within the country of origin (e.g., Mousavi 2021), and some measured the frequency of mask wearing (e.g., How long did you wear a face mask yesterday, Brankston 2020 [Brankston et al., 2020]). The terms ‘mask’ and ‘face covering’ was used interchangeably in some studies (Davis 2021; Milad 2021). The majority of studies did not specify how they defined masks or face coverings. For example, if they were measuring cloth face masks use or medical grade masks.

##### Determinants

There were 8 determinants analysed across the 23 studies, including worry and anxiety, perceived susceptibility, knowledge, perceived severity, self‐efficacy, perceived effectiveness and trust. Multiple determinants were reported within individual studies, for example, Mo 2021 reported on COVID‐related worry and anxiety, perceived susceptibility and severity, and self‐ efficacy. The most commonly reported determinants were trust (in government, neighbourhood, experts, etc.) (*n* = 6), and perceived susceptibility of COVID (*n* = 6), followed by perceived effectiveness of behaviour (n = 5), perceived severity (*n* = 5), and knowledge of COVID (*n* = 5). The least reported determinants included, COVID related worry and anxiety (*n* = 3) and self‐efficacy (*n* = 3).

Following assessment of the data, 10 studies were deemed suitable to include in the meta‐analysis (based of criteria detailed in ‘Methods: data synthesis’). These 10 studies reported on 3 determinants. A total of 16 studies were included in the narrative synthesis, reporting 6 determinants. Given the multiple determinants reported in individual studies, three studies were included in both the narrative synthesis and meta‐analysis (Kowalski 2020; Milad 2021; Mo 2021).

#### 6.1.3 Excluded studies

A total of three studies were excluded from this review, a list of which can found in the references.

### 6.2 Risk of bias in included studies

A detailed summary of risk of bias for the 23 included studies is shown in Table 3. All 23 studies were rated using the JBI tool for cross‐sectional studies (The Joanna Briggs Institute, 2017, 2020). Overall, 10 studies were rated low risk of bias, 10 unclear risk of bias, and 3 were rated as high risk bias. Those studies deemed high risk of risk predominately received this rating due to lack of detail on sample demographics and methodology (Iqbal 2021) (Iqbal & Younas, 2021); Varghese 2021; Zhong 2020 (Liang et al., 2022). One of these studies did not use a reliable or valid measure of mask wearing (Zhong 2020). Poor reporting of study design and methodology and lack of sample demographics made it difficult to determine representativeness in many of the studies rated as unclear risk of bias. In nine studies it was evident that the sample was not representative (Azlan et al., 2020); Davis 2021; Krupic 2021 [Dino et al., 2021]; Mousavi 2021; Muslih 2021; O'Brien 2021 [O'Brien William et al., 2021]; Rieger, 2020; Tran 2020; Zhong 2020).

**Table 3:** Quality appraisal of included studies.

Study	Were the criteria for inclusion in the sample clearly defined and adhered to?	Was the sample included in the study representative of the population of interest?	Were the determinants measured in a valid and reliable way?	Were the behaviours measured in a valid and reliable way?	Were confounding factors/covariates identified?	Were strategies to deal with confounding factors/covariates stated and used?	Was appropriate statistical analysis used?	Is there evidence of selective reporting?	Overall quality rating	Included determinants
Azlan (2020)	Yes	No	Yes—scale	Yes—scale	Yes	Yes	Yes	No	Low risk of bias	Knowledge
Brankston (2020)	Yes	Yes	Unclear	Unclear	Yes	Yes	Yes	No	Low risk of bias	Perceived susceptibility Perceived severity
Davis (2021)	Yes	No	Yes—scale	No—single item	Yes	Yes	No	No	Unclear risk of bias	Self‐efficacy Benefits
Goldberg (2020)	Yes	Unclear	Yes—scale	Yes—scale	Yes	Yes	Yes	No	Low risk of bias	Trust
Gray (2020)	Yes	Yes	Yes—scale	Yes—scale	Yes	Yes	Yes	No	Low risk of bias	Perceived effectiveness
Hao (2021)	Yes	Unclear	Yes—scale	Yes—single item	Yes	Yes	Yes	No	Low risk of bias	Trust
Iqbal (2021)	No	Unclear	Yes—single item	Yes—single item	Yes	No	Unclear	No	High risk of bias	Knowledge of COVID
Jang (2020)	Yes	Yes	Yes—scale	Unclear	Yes	Yes	Yes	No	Low risk of bias	Trust
Kowalski (2020)	Yes	Unclear	Yes—scale	Yes—single item	Yes	Yes	Yes	Yes	Low risk of bias	Anxiety about COVID Trust
Krupic (2021)	No	No	Yes—scale	Yes—scale	Yes	Yes	Yes	No	Low risk of bias	Anxiety about COVID
Lee (2020)	Yes	Unclear	Yes—scale	Yes—scale	Yes	Yes	Yes	No	Low risk of bias	Perceived susceptibility Perceived severity
Mahalik (2021)	Yes	Unclear	Yes—scale	Yes—scale	Yes	Yes	Yes	Unclear	Unclear risk of bias	Benefits
Milad (2021)	No	Unclear	Yes—scale	Yes—single item	Unclear	Yes	Yes	No	Unclear risk of bias	Perceived susceptibility Self‐efficacy
Mo (2021)	Yes	Yes	Yes—scale	Yes—scale	No	Yes	Yes	No	Unclear risk of bias	Worry about COVID Perceived susceptibility Perceived severity Self‐efficacy
Mousavi (2021)	Yes	No	Yes—single item	Unclear	Yes	Yes	Yes	Yes	Unclear risk of bias	Perceived severity
Muslih (2021)	Yes	No	Yes—scale	Yes—scale	Unclear	Unclear	Yes	No	Unclear risk of bias	Knowledge of COVID
O'Brien (2021)	No	No	Yes—scale	Yes—single item	Yes	No	Yes	No	Unclear risk of bias	Perceived susceptibility
Rieger (2020)	Yes	No	Unclear	Unclear	Yes	Yes	Yes	No	Unclear risk of bias	Perceived effectiveness
Stosic (2021)	No	Unclear	Yes—scale	Yes—scale	Yes	Yes	Unclear	Yes	Low risk of bias	Perceived effectiveness
Tran (2020)	Unclear	No	No—single item	Unclear	Yes	Yes	Yes	No	Unclear risk of bias	Perceived severity
Varghese (2021)	No	Unclear	Yes—single item	Yes—scale	No	Unclear	Yes	No	High risk of bias	Trust
Zhong (2020)	No	No	Yes—scale	No—single item	Yes	No	Yes	Yes	High risk of bias	Knowledge of COVID
Zhou (2021)	No	No	Yes—scale	Yes—scale	Yes	Yes	Yes	Yes	Unclear risk of bias	Knowledge of COVID

### 6.3 Effects of determinants

#### 6.3.1 Meta‐analysis

In total we analysed 11 effect sizes across 3 determinant groups, representing 25,725 participants. The summary effect of each determinant group can be seen Summary of findings Table 1 along with 95% CIs and heterogeneity statistics. As shown in the summary table, our analyses indicate a significant relationship between knowledge of COVID and mask wearing. There was no statistically significant relationship observed between perceived susceptibility, COVID‐related worry and anxiety and mask wearing. All data is reported in Tables 4, 5, 6.

**Table 4:** Knowledge of COVID and mask wearing.

Study	*n*	Description of determinant			Effect size
*Knowledge about COVID*					
Zhou (2021)	728	Knowledge about COVID‐19	Unadjusted	*r*	0.738
Muslih 2021	1033	Knowledge about COVID‐19	Unadjusted	OR	1.26
Iqbal (2021)	1789	Knowledge about COVID‐19	Unadjusted	Mean	8.15
Azlan (2020)	4850	Knowledge about COVID‐19	Unadjusted	Mean (SD)	10.3 (1.4) 10.6 (1.4)
Zhong (2020)	6910	Knowledge about COVID‐19	Unadjusted	Mean (SD)	10.8 (1.5) 9.3 (3.2)

Abbreviations: CI, confidence interval; OR, odds ratio.

**Table 5:** Worry and anxiety about COVID and mask wearing.

Study	*n*	Description of determinant			Effect size	CI
*Worry/anxiety about COVID*						
Kowalski (2020)	840	Coronavirus related anxiety	Unadjusted	*r*	0.2	
Krupic 2021	500	Coronavirus related anxiety	Unadjusted	*r*	−0.02	
Mo (2021)	4087	Worry of COVID‐19 infection	Unadjusted	OR	1.03	(1,1.05)

Abbreviations: CI, confidence interval; OR, odds ratio.

**Table 6:** Perceived susceptibility of COVID and mask wearing.

Study	*n*	Description of determinant			Effect size	CI
*Perceived susceptibility*						
Mo e2021	4087	Perceived susceptibility of Covid‐19	Unadjusted	OR	1.07	(1.02,1.11)
O'Brien (2021)	450	Perceived vulnerability to COVID‐19	Unadjusted	*r*	0.12	
Milad (2021)	451	Risk of exposure to COVID‐19	Unadjusted	*r*	0.15	

Abbreviations: CI, confidence interval; OR, odds ratio.

**Figure 2:**
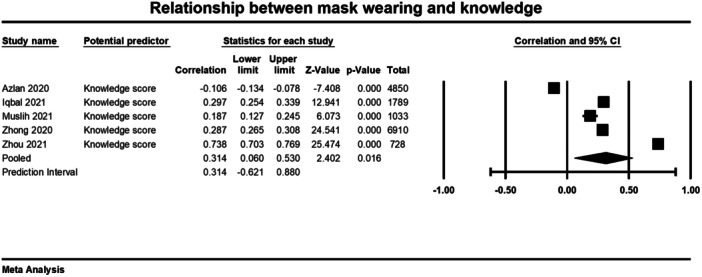
Relationship between mask wearing and knowledge. CI, confidence interval.

**Figure 3:**
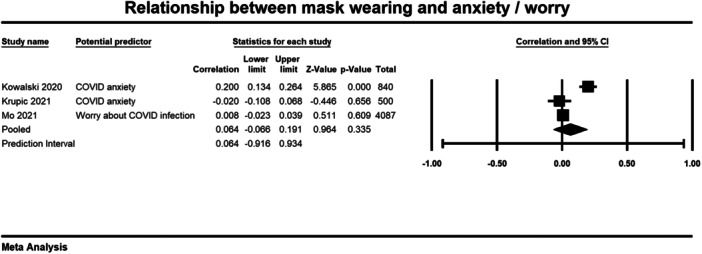
Relationship between mask wearing and wory and anxiety. CI, confidence interval.

**Figure 4:**
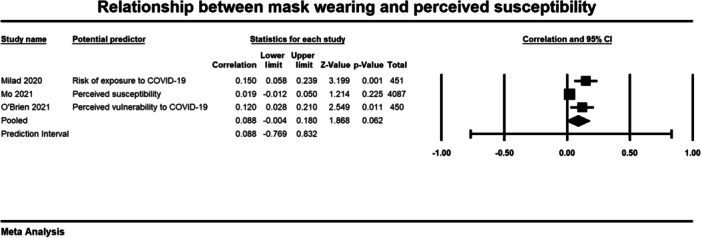
Relationship between mask wearing and perceived susceptibility. CI, confidence interval.

Below we present forest plots (Figures 2, 3, 4) for each determinant and interpret these findings further.

##### Knowledge

Five studies were included in the meta‐analysis that examined the relationship between knowledge of COVID and mask wearing (Azlan 2020; Iqbal 2021; Muslih 2021; Zhong 2020; Zhou 2021 [Min et al., 2021]). The average correlation coefficient across the 5 studies was moderate and significant (*r* = 0.314, 95% CI = 0.06, 0.530, *p* = 0.016) (Figure 2). There was significant heterogeneity across studies (*τ*² = 0.091; *Q* = 940.834, *df* = 4; *p* = < 0.001; *I*² = 100%). These results indicate that more knowledge of COVID was significantly correlated to mask wearing behaviour. However, there is a great deal of variation in the findings from individual studies and it is possible that this variation is caused by differences between the studies in the type of knowledge being assessed.

##### Worry and anxiety

The relationship between COVID‐related anxiety and worry and mask wearing was examined in three studies (Kowalski 2020; Krupic 2021; Mo 2021). The average correlation coefficient across the three studies was small and non‐significant (*r* = 0.064, 95% CI = −0.066, 0.191, *p* = 0.335) (Figure 3). There was significant heterogeneity across studies (*τ*² = 0.012; *Q* = 28.028, *df* = 2; *p* = < 0.001; *I*² = 93%). Therefore, experiencing more COVID‐related anxiety and worry was not significantly correlated to mask wearing behaviour.

##### Perceived susceptibility

Three studies examined the relationship between perceived susceptibility and mask wearing (Milad 2021; Mo 2021; O'Brien 2021). Similar to worry and anxiety, the average correlation coefficient across the three studies was small and non‐significant (*r* = 0.088, 95% CI = −0.004 to 0.180, *p* = 0.062) (Figure 4). There was significant heterogeneity across studies (*τ*² = 0.005; *Q* = 10.237, *df* = 2; *p* = 0.006; *I*² = 80%). The results indicate that perceived susceptibility of COVID was not significantly correlated to mask wearing behaviour.

#### 6.3.2 Synthesis of results

A total of 16 studies were included in the narrative synthesis. Details of the individual studies that contribute to this synthesis are shown in Table 2.

Our narrative synthesis found that perceived benefits and effectiveness of behaviours had the strongest association with mask wearing behaviour. Perceived susceptibility and perceived severity was also associated with mask wearing, although to a lesser degree. Trust and self‐efficacy had mixed results from the included studies.

##### Perceived susceptibility

Small but positive associations were reported in three studies (Brankston 2020; Lee 2020; Mo 2021) which examined the role of perceived susceptibility on mask wearing behaviour (Table 7). All studies reported greater levels of mask wearing in those who reported higher perceived susceptibility to COVID‐19. Small effects were found by Lee 2020 and Mo 2021 between mask wearing and perceived susceptibility. Lee 2020 included a smaller sample than Brankston 2020 and Mo 2021 (*n* = 973), due to lack of demographic details, it was unclear whether their sample of South Korean adults was representative. A larger effect was found by Brankston 2020, who examined predictors of mask use in a large (*n* = 4981) representative sample of Canadian adults.

**Table 7:** Perceived susceptibility and mask wearing.

Study ID	Determinant	Effect size (CI)	*n*
Lee (2020)	Perceived susceptibility	Multiple regression coefficient: 0.04 (−0.01 – 0.10)	973
Brankston (2020)	Perceived risk of contracting the virus	Multivariable regression coefficient: 1.31 (1.13–1.52)	4981
Mo (2021)	Perceived susceptibility	OR = 1.07 (1.02–1.11)	4087

Abbreviations: CI, confidence interval; OR, odds ratio.

##### Perceived severity

Five studies reported on the relationship between perceived severity and mask wearing (Brankston 2020; Lee 2020; Mo 2021; Mousavi 2021; Tran 2020) (Table 8). Similarly to perceived susceptibility, small but positive effect were found, with the five studies reporting greater levels of mask wearing in those with higher levels of perceived severity of COVID. Lee 2020 and Mousavi 2021 utilised smaller samples than in the three other studies, with 973 and 371 participants respectively. Larger effects were observed in Tran 2020 and Mousavi 2021, using Afghani and French samples respectively. Tran 2020 examined perceived severity and mask wearing among a large sample of adults with chronic conditions, 78% of their sample were female. While Mousavi 2021 had a smaller sample, who were predominately male (*n* = 72%) and younger (17–26 years 66%). Mo 2021 examined perceived severity among a large sample of pregnant women, and measured perceived severity of COVID‐19 for both the women and their new‐born baby.

**Table 8:** Perceived severity and mask wearing.

Study ID	Determinant	Effect size (CI)	*n*
Lee (2020)	Perceived severity	Multiple regression coefficient: 0.07 (0.02–0.13)	973
Mousavi (2021)	Low likelihood of survival if infected	OR = 1.89 (1.04–3.40)	317
Brankston (2020)	Perceived risk of contracting the virus	AOR: 1.61 (1.39–1.85)	4981
Tran (2020)	Feeling at risk of severe COVID	AOR: 1.93 (1.53–2.44)	7169
Mo (2021)	Perceived severity	OR = 1.05 (1.02–1.09)	4087

Abbreviations: AOR, adjusted odds ratio; CI, confidence interval; OR, odds ratio.

##### Self‐efficacy

Three studies reporting on the relationship between self‐efficacy and mask wearing behaviour (Davis 2021; Milad 2021; Mo 2021) (Table 9). The three studies had mixed results, with one reporting no effect (Mo 2021), and two reporting a positive effect (Davis 2021; Milad 2021). Davis 2021 examined self‐efficacy and mask wearing among university studies, with the final sample being predominately female (*n* = 73.4%). Larger effects between self‐efficacy and mask wearing were observed in Milad 2021, among a sample of the US general public. This study had a smaller sample and poor reporting of demographics meant that representativeness could not be determined.

**Table 9:** Self‐efficacy and mask wearing.

Study ID	Determinant	Effect size (CI)	*n*
Milad (2021)	Perceived control Self‐efficacy for mask wearing	*r* = 0.07, OR* = 1.29 *r* = 0.18, OR* = 1.94	451
Mo (2021)	Self‐efficacy	AOR: 1.00 (0.97–1.05)	4087
Davis (2021)	Behavioural confidence	Compliant individuals had higher behavioural confidence scores [*M*(SD) = 14.47(2.31)] vs. non‐compliant individuals [*M*(SD) = 11.08(4.06)], *d* = 1.03	596

Abbreviations: AOR, adjusted odds ratio; CI, confidence interval; OR, odds ratio.

##### Benefits/perceived effectiveness of behaviour

Perceived benefit and effectiveness of mask was measured in five studies (Davis 2021; Gray 2020 [Lesley et al., 2020]; Mahalik 2021; Rieger, 2020; Stosic 2021) (Table 10). Large effects were reported in two studies (Gray 2020; Mahalik 2021), with smaller effects reported in the remaining three studies (Davis 2021; Rieger, 2020; Stosic 2021). All studies reported that greater perceived benefits or effectiveness of masks was associated with more mask wearing behaviour, albeit to different strengths. In addition, there was heterogeneity among the samples. For example, Rieger, 2020 and Davis 2021 included a sample of university students, while Mahalik 2021 included only men. Both Stosic 2021 and Gray 2020 examined mask wearing behaviour in the general public, however, the demographics of participants were poorly reported in Stosic 2021.

**Table 10:** Benefits and perceived effectiveness and mask wearing.

Study ID	Determinant	Effect size (CI)	*n*
Stosic (2021)	Belief in mask effectiveness	AOR: 1.82 (1.68–2.00)	1050
Rieger (2020)	Perceived effectiveness of mask for protecting self Perceived effectiveness of mask for protecting others	Multiple regression coefficients: 0.65 0.57	201
Mahalik (2021)	Benefits of mask wearing	*r* = 0.51, OR* = 8.59	596
Gray (2020)	Perceived effectiveness of wearing: Surgical face masks Somewhat effective vs. not at all effective Very effective vs. not at all effective Cloth or home‐made face masks Somewhat effective vs. not at all effective Very effective vs. not at all effective Paper face masks Somewhat effective vs. not at all effective Very effective vs. not at all effective	ORs: 2.75 (1.58–4.78) 7.97 (4.35–14.59) 2.34 (1.55–3.53) 5.82 (3.00–11.58) 3.80 (2.60–5.55) 7.59 (3.80–15.16)	1015
Davis (2021)	Advantages	Compliant individuals had higher advantages score [*M*(SD) = 16.77(3.72)] vs. non‐compliant individuals [*M*(SD) = 13.35(6.81)], *d* = 0.62	596

Abbreviations: AOR, adjusted odds ratio; CI, confidence interval; OR, odds ratio.

##### Trust

Five studies were included in the synthesis of trust and mask wearing (Goldberg 2020; Hao 2021; Jang 2020 [Jang et al., 2020]; Kowalski 2020; Varghese 2021), with mixed results observed between them (Table 11). Larger positive effect sizes were reported in Varghese 2021 Kowalski 2020 and Jang 2020, while little to no effect was reported by Hao 2021 and Goldberg 2020. Goldberg 2020 and Hao 2021 included large US samples (*n* = 3933 and 1792, respectively). However, Hao 2021 found no effect between trusting people in the neighbourhood and mask wearing behaviour. Hao 2021 included a US sample, however there was poor reporting of demographics. Three studies reported larger effects between trust and mask wearing. Varghese 2021 measured trust in information from the World Health Organisation, in a large sample from 7 different countries, with equal representation from across the countries. However, it was rated high risk of bias due to poor reporting of demographics and methods. Those studies that reported larger effects, observed higher rates of mask wearing in those who reported greater levels of trust.

**Table 11:** Mask wearing and trust.

Study ID	Determinant	Effect size (CI)	*n*
Varghese (2021)	Level of trust	*r* = 0.12, OR* = 1.55	7000
Hao (2021)	Trust people in the neighbourhood	OR = 1.01	1792
Kowalski (2020)	Trust in the media	*r* = 0.14, OR* = 1.67	840
Goldberg (2020)	Trust in infectious disease experts	Regression coefficient for increase in mask wearing from before to after CDC recommendation = 0.07 (0.01–0.14)	3933
Kowalski (2020)	Trust in government	*r* = 0.22	840
Jang (2020)	Trust in government	AOR = 1.7999	1005

Abbreviations: AOR, adjusted odds ratio; CI, confidence interval; OR, odds ratio.

## 7 DISCUSSION

### 7.1 Summary of main results

This systematic review aimed to synthesise the evidence examining psychosocial factors that determine the uptake and adherence to mask and face covering behaviours for reducing the risk of infection or transmission of severe acute respiratory coronavirus 2 (SARS‐CoV‐2) in the general public.

The review forms part of the CoHeRe project (Hanratty et al., 2022). This interdisciplinary, multinational project has involved the development of an Evidence and Gap Map to identify and summarise current research on determinants of COVID‐19 protective behaviours, and a series of individual reviews examining the determinants of these specific behaviours (Hanratty et al., 2022).

This review provides one of the first studies to synthesise, using meta‐analyses and narrative summaries, evidence on the malleable factors that are most associated with mask and face covering behaviours. The focus on only malleable factors, excluding determinants such as demographic characteristics, is important, as it provides evidence to inform the development of interventions promoting face covering. Specifically, intervention targeted at malleable determinants of protective behaviours could be used as part of effective public health messages implemented to promote face covering behaviours in the context of potential future waves of COVID‐19, and other respiratory infections with pandemic potential.

A total of 23 studies were suitable for inclusion in the review, representing 54,401 participants. The majority of included studies were online, cross‐sectional studies, with the majority being published in the United States (*n* = 4) or China (*n* = 3). Thirteen studies were published in 2021, within the first 12 months of the COVID‐19 pandemic being declared.

Across all 23 included studies the most common malleable determinants of face covering were perceived susceptibility (*n* = 6 studies, 26%), and trust (*n* = 6 studies, 26%). Smaller numbers of studies examined determinants such as self‐efficacy (*n* = 3, 13%) and worry and anxiety (*n* = 3, 13%). Across the studies included in the meta‐analysis, knowledge was the most common malleable determinant (*n* = 5). In the narrative synthesis, trust was the most commonly reported determinant (*n* = 6).

Overall findings from the meta‐analysis indicate that knowledge of COVID was the malleable determinant most associated with mask wearing behaviour. With no association observed between perceived susceptibility, COVID‐related worry and anxiety and mask wearing. The narrative synthesis found that perceived susceptibility and perceived severity were associated with mask wearing, albeit with small effect. However, trust and self‐efficacy had mixed results from the included studies. In the narrative synthesis the strongest association was found between perceived benefits and effectiveness of behaviours and mask wearing behaviour.

Findings from the meta‐analysis and narrative synthesis did therefore show some agreement, particularly related to the lack of association between face covering and perceived susceptibility. Although agreement was observed, it should also be noted that it is difficult to make any strong inferences for some determinants, partly due to the differences in measurement and samples in some of the studies in the meta‐analysis and the narrative synthesis (for example, trust, perceived benefits). Furthermore, due to the small number of studies included in the meta‐analysis, results should be interpreted cautiously.

It is important to note that the meta‐analyses presented in this review have a high degree of heterogeneity. This heterogeneity could be a result of variation in the measurement or operational definition of the determinants, or variation in the measurement or operational definition of face covering, or variation in the timing of the study in relation to government‐led initiatives or mandates within each country. Furthermore, the evidence presented in the review is drawn from cross‐sectional studies, which prevents any conclusions being drawn that go beyond associations between variables. In other words, the review does not help us to understand how change in the determinants might be related to change in handwashing behaviour. This is a gap for further research.

### 7.2 Overall completeness and applicability of evidence

To the best of our knowledge, the evidence presented in this review represents the entirety of research to date (completed searches October 2021) on malleable determinants of mask wearing as a COVID‐related behaviour. During this review, we followed a pre‐registered peer‐reviewed protocol that was developed in consultation with expert stakeholders and methods experts. A comprehensive search was conducted to identify relevant studies and a team of experts and reviewers worked independently to select studies using the predetermined eligibility criteria and extract outcome data using a standardised data extraction form.

Ten studies (25,725 participants) were suitable for pooling of data in the meta‐analysis.

Samples from 9 countries were represented in the 23 included studies. The majority of these being from the USA (*n* = 4) and China (*n* = 3). Given that COVID‐19 is a global pandemic the narrower geographical coverage of the studies may limit the applicability of the evidence.

This was a large review examining data from a total of 54,401 participants across the 23 studies on one COVID‐related behaviour. The research on COVID‐19 has been published at a rapid rate since the beginning of the pandemic. A rapid review conducted in 2020 as part of the CoHeRe project (Hanratty et al., 2021), included 54 studies looking at 9 different COVID related behaviours. This review included 23 studies looking at mask wearing alone, evidencing the rapidly increasing volume of COVID‐related research. This review provides one of the first studies to synthesis evidence on the malleable factors that are most associated with mask wearing, and is one of a series of reviews on 9 different COVID‐related behaviours. This review and subsequent reviews are highly applicable to those involved in the development and implementation of public health decisions, interventions, and messaging to promote health behaviours in the context of COVID‐19, and other respiratory infections.

### 7.3 Quality of the evidence

The majority of the included studies were of fair methodological quality. However, a number of studies [*n* = 3 (13%)], were assessed as being of low quality due to the presence of methodological limitations, primarily, lack of clarity over recruitment and methods (Table 3).

### 7.4 Potential biases in the review process

To limit potential bias, a systematic approach, which included input from an information retrieval specialist, was used to plan and conduct the searches and the study identification process.

Searches also included information sources such as trial registers and repositories, which were used to identify recent and rapidly emerging evidence. Other strengths include the extensive use of stakeholder involvement via advisory panel input, and through participation of the Cochrane Crowd, who contributed to the screening of a large number of potential records for inclusion. Screening was completed by three reviewers independently. In addition, 20% of all studies were checked by a second author throughout the screening and extraction process.

### 7.5 Agreements and disagreements with other studies or reviews

There are a number of related published and ongoing reviews on determinants of COVID‐19 health‐related behaviours but none with the broad scope of this review. A recently published review by (Liang et al., 2022) examined the psychosocial determinant of hand hygiene, mask wearing and physical distancing. They included 24 studies examining face mask wearing and applied the Risk, Attitudes, Norms, Abilities, and Self‐Regulation (RANAS) model when determining determinants of interest. They found that knowledge and benefits, and effectiveness of preventive behaviour were significantly associated with facemask wearing, whereas perceived susceptibility and severity was not (Liang et al., 2022). Therefore the finding from this current review concur and further add to those found by (Liang et al., 2022).

## 8 AUTHORS’ CONCLUSIONS

### 8.1 Implications for practice and policy

The findings from this review indicate that knowledge of COVID and perceived benefits are the determinants most associated with mask wearing behaviour. While determinants like perceived susceptibility and COVID‐related worry and anxiety have little to no effect on face covering behaviour. An understanding of how these malleable determinants impact mask wearing behaviour provides evidence to inform the development of future interventions, and public health campaigns. Moreover, this evidence provides important insights regarding the determinants of mask wearing for potential future waves of COVID‐19, and other respiratory infections.

### 8.2 Implications for research

The volume of research on COVID has rapidly increased from the beginning of the pandemic, and continues to emerge. Increased demand to understand the determinants of COVID‐19‐related behaviour has resulted studies being completed rapidly, often at the expense of the quality of the research (Park et al., 2021). Other studies have similarly pointed to the need for well‐designed, good quality studies (Park et al., 2021), on the determinants of COVID related behaviour. In addition, the majority of our studies were from high‐income countries, largely the USA and China. COVID‐19 is a global pandemic, thus we need to understand how and if the determinants of behaviour vary globally. Finally, the most commonly reported determinants were perceived susceptibility and trust. Our research has shown these to have little to no effect on mask wearing, albeit these results must be interpreted cautiously. Determinants such as knowledge were less commonly reported however had a larger effect on mask wearing. Of the 23 included studies, none considered determinants such as social norms, knowledge of behaviours or motivations, despite evidence suggesting these may be associated with mask wearing. These determinants should be considered further.

## CONTRIBUTIONS OF AUTHORS

This review was undertaken by a team with substantial expertise in systematic reviews, health behaviour and infectious diseases. Professor Martin Dempster, Principal Investigator (PI) of the project had overall responsibility for its conduct and delivery. Dr Sean O'Connor, Dr Rachel Leonard and Dr Jennifer Hanratty was responsible for the day‐to‐day operation of the review, led screening, data extraction, quality assessment and reporting. Dr Ciara Keenan acted as an information retrieval specialist, designed and conducted the searches, and contributed to screening and data extraction.

Ariana Axiaq, Yuan Chi, Victoria Hawkins, Kerry Campbell, Ceri Welsh, Anna Volz and Jenny Ferguson contributed to screening and data‐extraction. Professor Miller acted as advisor on evidence synthesis methodology. Dr Bradley was the content expert on communicable diseases and reviewed and commented on drafts.

Dr Jennifer Hanratty is a psychologist and expert in evidence synthesis. She has worked in evidence synthesis since 2012 and published reviews with Campbell, Cochrane and NIHR Health Technology

Assessment among others, was editor with Campbell Education Co‐ordinating group, Fellow with Campbell UK and Ireland and an invited member of the international advisory board for Evidence Synthesis Ireland.

Dr Sean O'Connor is a Physiotherapist and an experienced health care researcher. He has undertaken a number of systematic reviews and studies related to behavioural interventions, including in the context of COVID‐19. He has an extensive knowledge of theory‐based implementation models for maximising integration of evidence into practice, systematic review methods including methodological quality/risk of bias assessment and the examination of stakeholder perspectives in healthcare delivery.

Dr Rachel Leonard is a Social Worker and an experienced health care researcher. She has experience of conducting and leading on a number of systematic reviews, meta‐analyses, and studies related to health interventions.

Dr Ciara Keenan is a methods editor and information retrieval specialist for the Campbell collaboration. She has considerable experience conducting and leading the creation of EGMs and systematic reviews.

Ariana Axiaq is a third year medical student with an interest in health promotion and extensive research experience in thematic analyses and systematic reviews.

Yuan Chi is Cochrane Information Specialist, Founder CEO of Yealth Technology, core team member with Cochrane COVID‐19 Study Register (https://covid-19.cochrane.org/), and the only Chinese executive team member for COVID19 Recommendation Map. She has 8 years experience on evidence synthesis, assisted 50+ international projects, resulting in 11 authorships and 28 recommendations from Cochrane editors, information specialists and authors.

Dr Jenny Ferguson is a researcher with a background in the use of technology to provide training in social communication to parents of autistic children. She has experience in assisting and conducting Systematic Literature Reviews across a number of related topics.

Victoria Hawkins is a Masters student in the School of Psyhcology, QUB.

Kerry Campbell is a PhD student in the School of Psyhcology, QUB.

Ceri Welsh is a PhD student in the School of Psyhcology, QUB.

Anna Volz is a third year undergraduate psychology student.

Professor Sarah Miller is Director of Campbell UK & Ireland. She is co‐chair and co‐editor of the Campbell Education Coordinating Group.

Dr Bradley is a consultant in public health medicine and clinical lecturer in public health and was consultant in health protection (communicable disease control) before taking up his current post. His publishing record includes several systematic reviews and studies of healthcare‐related behaviour. He is a member of the Northern Ireland COVID‐19 Modelling and Behaviour Change Groups

Professor Dempster, is a registered Health Psychologist and Chartered Statistician, with over 20 years’ experience in conducting research on the determinants of behaviour change. He has published 14 reviews, including reviews of effectiveness and reviews of covariates. He is currently a member of the Northern Ireland Public Health Agency COVID‐19 Behaviour Change Group.

- Content: Bradley, Dempster, Hanratty, Miller, Keenan, O'Connor, Leonard
- Systematic review methods: Hanratty, Miller, Dempster, Keenan, O'Connor, Leonard
- Statistical analysis: Dempster, Miller, Hanratty, Keenan, O'Connor, Leonard, Ferguson, Axiaq, Chi,Volz, Welsh, Campbell, Hawkins
- Qualitative Evidence Synthesis: n/a
- Information retrieval: Hanratty, Keenan, O'Connor

## DECLARATIONS OF INTEREST

None of the review team have any present or past affiliations or other involvement in any organisation or entity with an interest in the review's findings that might lead to a real or perceived conflict of interest.

## PLANS FOR UPDATING THIS REVIEW

This review will not be updated by the project team as the end of our funding period is on 18th October 2022. Teams interested in building on the review or contributing to updating beyond the project end date are encouraged to contact the corresponding author.

## SOURCES OF SUPPORT

### Internal sources

- No sources of support provided

### External sources

- No sources of support provided

### PEER REVIEW

The peer review history for this article is available at https://www.webofscience.com/api/gateway/wos/peer-review/10.1002/cl2.1422.

## Supporting information

Supporting information.

Supporting information.

## Data Availability

n/a
