# Supplementary material for: Psychological and psychosocial determinants of COVID related face covering behaviours: A systematic review
Source: Campbell Syst Rev. 2024 Jul 20;20(3):e1422. doi: 10.1002/cl2.1422 (PMC11260276; doi:10.1002/cl2.1422)
Supplement: Supplementary file 1 — Supporting information. [file CL2-20-e1422-s002.docx]

**Appendix 1. Data extraction Sheet for COHeRe Review**

| Study ID | | First author + year | |
| --- | --- | --- | --- |
| **Behaviour** | | | |
| Preventative Behaviour | | List  **Behaviour**  **Description of behaviours**  Handwashing  Washing hands more frequently with soap and water or the use of hand sanitizer if handwashing facilities are not available  Masks /face covering  Wearing any type of mask or face covering. This can include medical grade masks, face shields, homemade masks, covering face with a scarf etc.  Physical Distancing  Maintaining the recommended distance from others when physically present. The recommended distance varies by setting but is typically in the region of 1 to 3 meters.  Social Distancing  Minimising social contact with those outside of your own household. This is a very broad category and includes working from home, avoiding crowded places, only leaving home when necessary (e.g. to purchase food or medicines) and not socialising with others in your own home or garden.  Isolation /quarantine  Self-isolation and/or quarantine refers to keeping separate from all other people either because you have or are suspected to have the virus. Self-isolation is typically voluntary but often recommended by the government/health authorities. Quarantine is typically enforced in either a mandated setting, one's own home, or temporary accommodation for those in travelling away from home.  Respiratory hygiene/ etiquette  Includes tissue hygiene, which means using a tissue to cover nose and mouth when coughing, sneezing or blowing your nose and immediately disposing of the tissue. When tissues are not available coughing/sneezing into your elbow and not your hands.  Cleaning surfaces  Disinfecting high touch surfaces in home and office/retail/public spaces or items brought into the home.  Avoiding t-zone  Avoiding touching your face specifically the t-zone; eyes, nose & mouth  Other  Other analogous relevant behaviours or aggregate measures of multiple relevant behaviours | |
| Description | | Brief description of behaviour as reported in the study | |
| When? | | Reporting time frame  Retrospective reporting  Current behaviour  diary methods, reporting on today or observation of behaviour in real time.  Prospective/hypothetical  report on intended or anticipated future behaviour | |
| Who? | | Who collected the information?  1. Self-report  2. Family-report  3. Clinician/medical profession  4. Independent observer  5. Other  Not Specified | |
| Type? | | Type of Measurement  1. Clinical (direct assessment perhaps through administrative recored)  2. Self/other Report (questionnaire or survey)  3. Physical Specimens  4. Observation  5. Structured Interview  6. Other (please specify)  7. Not Specified | |
| Quality? | | Were the measurement tools reported as reliable/valid?  Yes - reliable  No - not reliable  Not reported  Not applicable | |
| Direction? | | Direction of scales  Higher score is good/more is better  Lower score is good/less is better  NA | |
| **Determinant** | | | |
| Description | | Brief description of determinant as reported in the study | |
| When? | | Reporting time frame (list as above) | |
| Who? | | Who collected the information? (list as above) | |
| Type? | | Type of Measurement (list as above) | |
| Quality? | | Were the measurement tools reported as reliable/valid? (list as above) | |
| Direction? | | Direction of scales (list as above) | |
| Adjusted or unadjusted? | | Speify weather the data is adjusted or unadjusted and what variables were accounted for in the analysis | |
| **Behaviour/Determinant relationship quantitative data** | | | |
| Summary | Description of findings | | Narrative description of findings (copy paste from paper or summarise in own words) |
| n | n | | sample size for this specific analysis |
| Data - complete relevant fields only | r | | correl |
|  | d | | cohens d |
|  | OR | | odds ratio or adjusted odds ratio |
|  | B | | regression coefficient (adjusted or not) |
|  | SDIV | | Standard deviation of IV |
|  | SDDV | | Standard deviation of DV |
|  | p | | P value reported |
|  | Lower CI | | Lower confidence interval |
|  | Upper CI | | Upper confidence interval |
|  | N w | | N with determinant |
|  | N wo | | N without determinant |
|  | E w | | Events with determinant |
|  | E wo | | Events without determinant |
|  | F | | F |
|  | df2F | | df2F |
|  | t | | t |
|  | dft | | dft |
|  | Mean1 | | Mean1 |
|  | Mean2 | | Mean2 |
|  | SE1 | | SE1 |
|  | SE2 | | SE2 |
|  | SD1 | | SD1 |
|  | SD2 | | SD2 |
|  | n1 | | n1 |
|  | n2 | | n2 |

**Appendix 2. Modifications to the JBI tool for assessing risk of bias/quality in cross-sectional studies**

| **Original tool** | **Modified/additional items** |
| --- | --- |
| 1. Were the criteria for inclusion in the sample clearly defined? | 1a. Were the criteria for inclusion in the sample clearly defined **and adhered to**? |
|  | 1b. Was the sample randomly selected from the defined population? |
| 2. Were the study subjects and the setting described in detail? | 2. Was the sample included in the study representative of the population of interest? |
| 3. Was the exposure measured in a valid and reliable way? | 3. Were the **determinants** measured in a valid and reliable way? |
| 4. Were objective, standard criteria used for measurement of the condition? | 4a.. Were objective, standard criteria used for measurement of the **behaviours** of interest? |
|  | 4b. Were the **behaviours** measured in a valid and reliable way? |
| 5. Were confounding factors identified? | 5. Were confounding factors /**coviariates** identified? |
| 6. Were strategies to deal with confounding factors stated? | 6. Were strategies to deal with confounding factors/ **covariates** stated **and used**? |
| 7. Were the outcomes measured in a valid and reliable way? | no change |
| 8. Was appropriate statistical analysis used? | no change |
